# An FPGA-Embedded Brain-Computer Interface System to Support Individual Autonomy in Locked-In Individuals

**DOI:** 10.3390/s22010318

**Published:** 2022-01-01

**Authors:** Arrigo Palumbo, Nicola Ielpo, Barbara Calabrese

**Affiliations:** Department of Medical and Surgical Sciences, Magna Graecia University, Viale Europa, 88100 Catanzaro, Italy; palumbo@unicz.it (A.P.); ielpon@unicz.it (N.I.)

**Keywords:** embedded systems, brain-computer interface, EEG, FPGA

## Abstract

Brain-computer interfaces (BCI) can detect specific EEG patterns and translate them into control signals for external devices by providing people suffering from severe motor disabilities with an alternative/additional channel to communicate and interact with the outer world. Many EEG-based BCIs rely on the P300 event-related potentials, mainly because they require training times for the user relatively short and provide higher selection speed. This paper proposes a P300-based portable embedded BCI system realized through an embedded hardware platform based on FPGA (field-programmable gate array), ensuring flexibility, reliability, and high-performance features. The system acquires EEG data during user visual stimulation and processes them in a real-time way to correctly detect and recognize the EEG features. The BCI system is designed to allow to user to perform communication and domotic controls.

## 1. Introduction

The purpose of the BCI research is the realization of a new assistive communication and control technology for people with severe neuromuscular disabilities [1,2,3], such as amyotrophic lateral sclerosis (ALS), spinal cord injury, stroke, multiple sclerosis, and muscular dystrophies [4,5,6,7,8,9,10,11,12,13]. Therefore a potential group of BCI users consists of people who cannot activate any muscle despite having an adequate cognitive function (locked-in syndrome). In addition, in the last years, this technology has been used in cognitive studies, i.e., for clinical diagnosis and prognosis for patients with disorders of consciousness [14]. In 2012, Wolpaw tried to provide a definition that is exhaustive and complete [15]: A BCI is a system that measures central nervous system (CNS) activity and converts it into an artificial output that replaces, restores, enhances, supplements, or improves natural CNS output and thereby changes the ongoing interactions between the CNS and its external or internal environment. In general, the CNS function responds to events in the outside world or the body by producing natural outputs (neuromuscular or hormonal) that meet the organism’s needs. The phenomena that occur continuously in the CNS (electrophysiological, neurochemical, and metabolic) can be quantified by monitoring electric or magnetic fields or other parameters using sensors on the scalp, the brain’s surface, or within the brain. A BCI, then, acquires the brain signals, analyses them to extract specific measures (or features) that correlate with the user’s intent, and converts (or translates) these features into commands that control the application devices [16,17]. The brain signals can be acquired through different electrophysiological methods, but electroencephalography (EEG) is the most used non-invasive signal acquisition method for BCI [18].

Different components of the EEG signal could be used to control BCI systems, such as sensorimotor rhythms [19,20,21], slow cortical potentials [22], P300 event-related potentials [23,24], and steady-state visual evoked potentials (SSVEP) [25,26], but the P300 and SSVEP-based BCIs turn out to be the most effective for communication and control applications. They allow users to select different characters or icons relatively quickly without requiring intensive training. Specifically, SSVEP is a visual evoked potential (VEP) consisting of a visual cortical response evoked by repetitive stimuli with a constant frequency on the central retina. For example, when the retina is excited by a visual stimulus at frequencies between 3.5 and 7.5 Hz, the brain yields an electrical activity at the same and different frequency of the visual stimulation. The main disadvantages of the SSVEP paradigm are here reported. First, there could be the risk of inducing photo epileptic seizures for stimulation frequencies in the 15–25 Hz band. Secondly, SSVEP-based BCIs perform much worse if the BCI system should discriminate when the subject does nothing versus attending to the SSVEP stimulus. This problem is known as the zero-class problem and is a severe issue in real-world BCI systems. P300, instead, can be evoked in nearly all subjects and be easily elicited differently from other visual evoked potentials.

Farwell and Donchin introduced the first P300-based BCI system in 1988; their goal was to allow paralyzed people to communicate simple messages using their system [23]. They proposed a design in which a 6 ∗ 6 matrix of letters and other commands was presented to the user. The stimulation consisted of flashing the rows and columns of the matrix in random order; the user had to focus on a letter and mentally count the number of times it was illuminated. This is defined as the “oddball” paradigm. After several repetitions, the computer was able to identify the row and column that had elicited the P300 component. Then, the letter that the user wanted to select could be obtained by the intersection of the two. Over the years, several groups have perfected this system through the use of alternative EEG registration sites, signal-processing methods, and stimulus presentation parameters and formats to improve the speed, accuracy, capacity, and clinical practicality of the P300-based BCI systems, to make them a valid option of communication and control for people with severe motor disabilities [27,28,29,30,31,32,33,34]. These BCI systems are based on the use of personal computers. They, however, are neither compact nor portable because the EEG signal is amplified and conditioned with commercial amplifiers, and the algorithms for features extraction and classification work on personal computers; in this way, these systems cannot be used conveniently in hospitals or at home [35,36]. Instead, parameters such as energy consumption, size, robustness, portability, and reconfiguration must be considered to make the BCI system an effective communication and control device. In recent years some embedded BCI system designs have been proposed. Gao et al. presented an SSVEP-based BCI system to control environmental devices, such as TV, videotape recorders, or air-conditioners [37]. Compared to their previous PC-based BCI system, they used a new stimulator composed of 48 green LEDs, whose blinking frequency is controlled by a programmable logic device, a trainable infrared remote-controller a DSP system for all processing and control functions. The results obtained on a subject show a transfer rate up to 68 bits per minute, an average accuracy of 87.5%, and an average time for one target selection of 3.8 s. Lin et al. proposed a real-time wireless embedded EEG-based BCI system for real-time drivers’ drowsiness detection and warning [38]. The system consists of a four-channel signal acquisition and amplification unit, a wireless data transmission unit, a dual-core embedded system, a host system for data storage and real-time display, and a warning device. The system was tested on five subjects, and it was achieved an average accuracy of 74.6%. Garcia et al. presented a versatile hardware platform to produce a small, autonomous, and configurable BCI platform adaptable to the user’s needs [39]. It consists of three modules: (i) an EEG amplifier and digitizer, (ii) a micro-controller to handle the transfer of the EEG samples in real-time to the computer, and (iii) a communication module that uses the Zigbee or Bluetooth protocols. However, signal-processing algorithms have been implemented on personal computers. In 2010 it was presented an embedded SSVEP-based BCI based on a low-cost field-programmable gate array (FPGA) [40]. The system includes a customized light-emitting diode (LED) stimulation panel, an SSVEP acquisition circuit, an FPGA-based real-time signal processor, a radio-frequency (RF) command transmitter-receiver circuit, and a bio-feedback voice-output circuit and allows users to control multimedia devices. The system has been tested on seven subjects (ages 23 to 32); it was obtained an accuracy of 89.29% and an information transfer rate of 24.67 bits per minute. Joshi et al. presented a portable and economic mu rhythm-based BCI accomplished using a programmable system on chip (PSoC, Cypress Semiconductor) [41]. Through the motor imagery, the user must move a cursor located on the center of a screen to targets in the top and bottom. The system was tested on three subjects for two weeks; it was obtained an average accuracy of 70% and a communication bit rate of up to 7 bits per minute on the final day.

This paper describes an embedded BCI system based on the acquisition and processing of EEG signals aiming to extract and recognize P300 components elicited through the oddball paradigm. An FPGA-based device was selected among different possible embedded solutions for the system’s development since it offers several advantages, such as flexibility, reliability, and high-performance features [42,43]. In particular, since the FPGA chip contains reconfigurable gate arrays and embedded memory, it is suitable for rapidly implementing a digital signal-processing algorithm. Moreover, the use of parallel architectures increases system performance by processing high-volume data more efficiently and performing multiple tasks simultaneously. The event-related potential was chosen as the control characteristics of our BCI system due to the relatively short training times required to the user and the increased speed, which allows the selection of one among several options proposed concerning the sensorimotor rhythms. SSVEPs also enable the user to select a choice between different possibilities quickly without training. However, as reported in the work of [44], P300-based systems allow higher classification accuracy than SSVEP-based BCI systems. Therefore, P300 potential has been preferred.

The paper is organized as follows: Section 2 describes the architecture and the main functionalities of the FPGA-based brain-computer interface system; Section 3 presents the main results of the experimental tests. Finally, Section 4 concludes the paper, highlighting the innovative features of our system.

## 2. Methods

**Figure 1:**
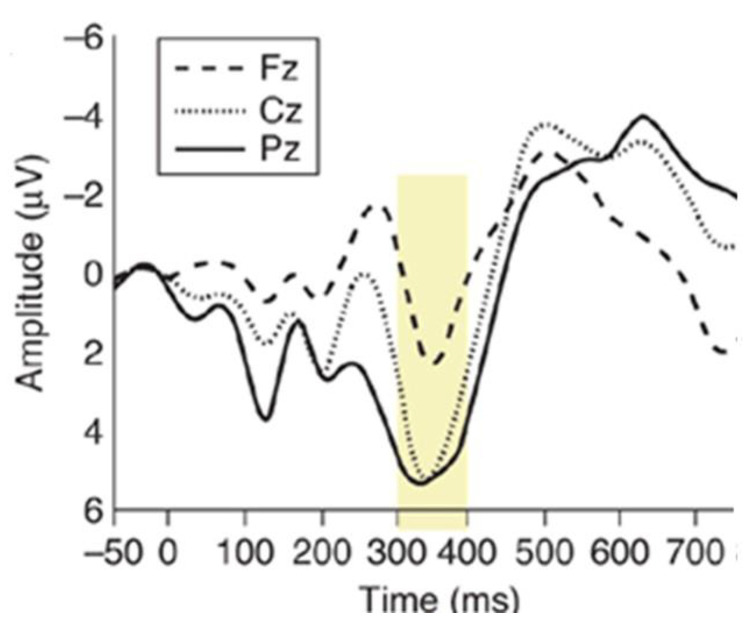
P300 component: the yellow box highlights the P300 component for averaged waveforms from different electrode positions. P300 amplitude decreases as the electrode site moves from anterior (Fz) to posterior (Pz).

The P300 event-related potential (ERP) is a cognitive potential that occurs when the subject recognizes a rare or relevant stimulus (target), auditory, visual, or somatosensory, within a train of frequent or irrelevant stimuli (non-target) [45]. An example of a stimulation paradigm is the oddball paradigm. In this paradigm, the users are subjected to events that can be classified into two distinct categories. Events that belong to one of two types rarely occur. When the rare event is presented to the user, it elicits the P300 potential. In addition to the oddball paradigm, i.e., the single-stimulus and three-stimulus paradigms can be used. P300 consists of a positive deflection in the EEG, which occurs 250–400 ms after the onset of the target stimulus (Figure 1).

The P300 potential has an amplitude lower or similar to the EEG background activity; for this reason, it is necessary to synchronize media from different epochs relating to the target stimuli to distinguish the P300 potential from regular EEG activity. The amplitude of the P300 component is inversely proportional to the frequency with which the rare impulse occurs; it is also influenced by the number of concurrent activities performed by the user and by changes in the probability of the target stimulus; for example, if two target stimuli occur consecutively, the amplitude of the P300 potential decreases after the first rare stimulus [46].

**Figure 2:**
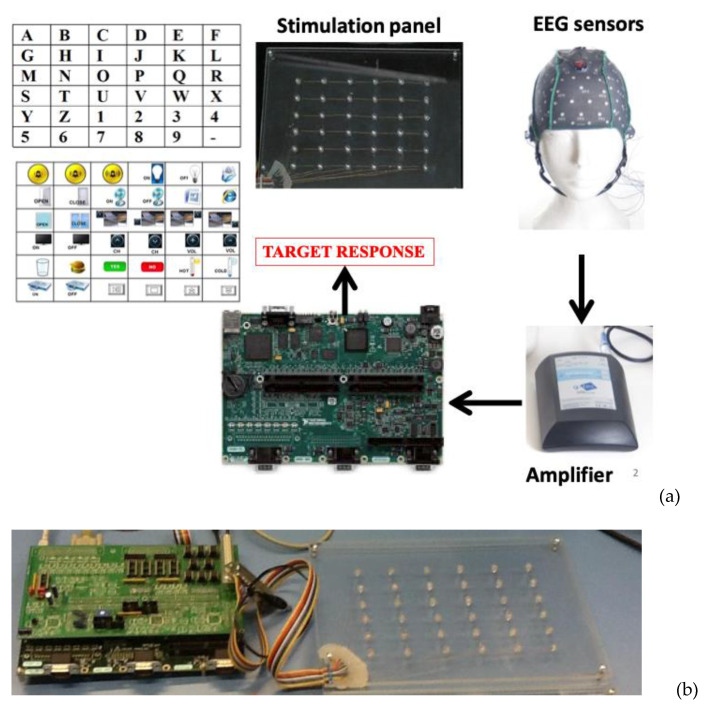
(**a**) Hardware modules of the proposed FPGA-based BCI system; (**b**) stimulation panel and embedded-FPGA-based board.

Figure 2a shows the architecture of the proposed FPGA-embedded BCI system, which includes the following hardware modules: (i) a custom visual stimulator; (ii) a commercial certified EEG amplifier (g.Mobilab+, g.TEC Graz, Austria); (iii) an embedded hardware platform for processing and systems control (Single-Board RIO, National Instruments, Austin, TX, USA). In Figure 2b, it is possible to see the connections between the stimulation panel and the embedded FPGA-based board.

The stimulation panel contains 36 light-emitting diodes (LEDs) organized in a matrix with six rows and six columns. The LEDs’ flicking is managed according to the oddball stimulation paradigm by the embedded platform to evoke the P300 response.

**Figure 3:**
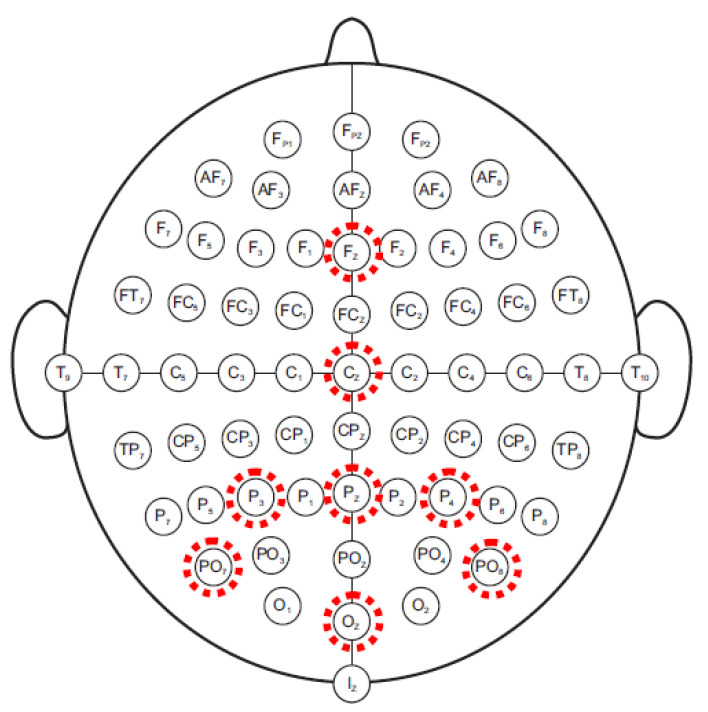
Electrode montage for P300 protocol.

EEG amplifier acquires signals coming from eight-sintered silver/silver chloride electrodes (mounted on an EEG cap) placed according to the International 10/20 System at Fz, Cz, Oz, Pz, P3, P4, PO7, and PO8. All electrodes were referenced to the right earlobe and grounded to the left mastoid. The electrodes that P300 is typically recorded from are illustrated in Figure 3: the P300 component is more evident in the occipital and parietal regions. The eight channels were amplified, band-pass filtered between 0.5 and 100 Hz, and digitized (with a 16-bit resolution) at a 256 Hz sampling rate. Through a serial interface (RS-232), EEG data are transferred to the embedded platform.

The embedded platform used for developing the BCI system is an integrated acquisition and processing system based on FPGA technology. The single-board RIO system is a low-cost deployment solution based on National Instruments Reconfigurable I/O (RIO) technology. On a single board, it integrates a real-time processor for reliable stand-alone operation and signal-processing, reconfigurable FPGA for custom I/O timing and processing, and analog and digital I/O. The real-time processor is connected to the reconfigurable FPGA Xilinx (Spartan Family) through an internal high-speed PCI bus. The board features an industrial 400 MHz Freescale MPC5200 processor that determines LabVIEW Real-Time applications on the reliable Wind River VxWorks real-time operating system. In addition, FPGA is directly connected to different I/O modules.

The embedded platform can be programmed using the LabVIEW graphical programming language and two specific add-on modules (LabVIEW Real-Time Module to create applications that can run in the embedded processor and the LabVIEW FPGA Module to program the FPGA integrated).

**Figure 4:**
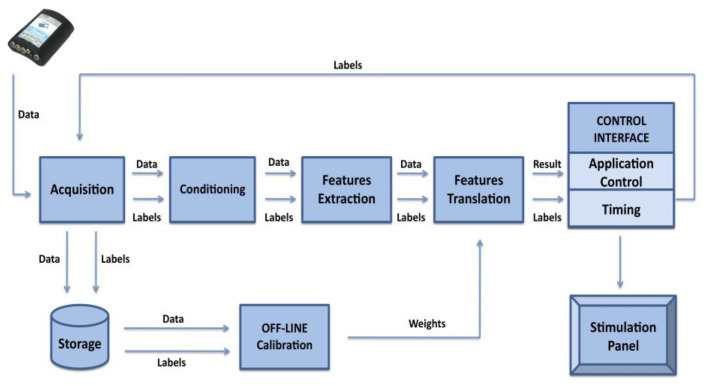
Block diagram of the FPGA-based system.

The embedded hardware system performs all signal-processing steps needed to extract the P300 response. In particular, the implemented hardware blocks include conditioning (pre-processing), feature extraction, and feature translation (classification) (see Figure 4). Moreover, the embedded system manages the data acquisition from the EEG amplifier, the timing of stimulation, and gives visual feedback to the user relative to the classification results at the end of the classification process. In addition to displaying classification results, the embedded system can operate electrical/electronic devices, i.e., for domestic applications, using digital I/O lines. A detailed description of different BCI blocks is reported in the following sections.

### 2.1. Visual Stimulation

The stimulation interface panel consists of 36 LEDs arranged in a six by six matrix. The panel’s physical size is 25 cm by 17 cm. The LEDs are mounted on a Plexiglas plate and driven through a custom hardware module. LEDs have been chosen because they have several advantages over traditional devices used for visual stimulation in neuroscience (e.g., LCD screen): they are small, relatively stable, cheap, demand little energy to be driven, and have low electromagnetic emissions. Moreover, due to their on/off switching response, LEDs are suitable for displaying precise temporal patterns of stimulation [47]. In particular, green LEDs have been used because some studies [48] have demonstrated that a green/blue flicker matrix can be associated with better performances in a P300 BCI, maintaining safe conditions for the user.

**Figure 5:**
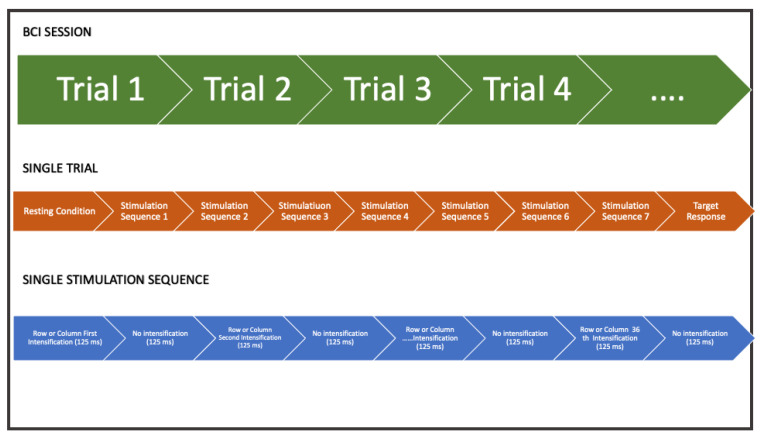
An example of timing duration of experimental protocol for single trial and all trials of the individual subject: A single BCI session is formed by a certain number of trials according to the user application. The user is stimulated with several stimulation sequences (for example, 7). In a single stimulation sequence, all rows and columns are intensified.

**Figure 6:**
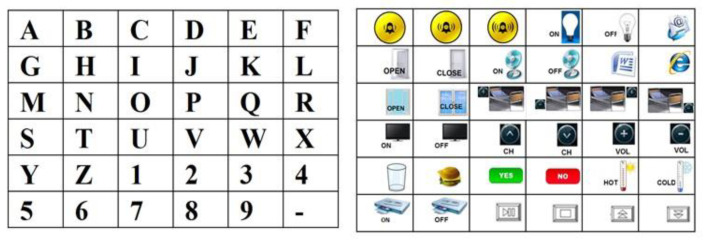
Communicator sheets for communication and domotic applications.

The LEDs driving module provides a robust and high current capacity to the LEDs matrix. The led driver receives pulse signals from the digital ports of the embedded hardware system, such that LEDs can be turned on and off according to the stimulation timing protocol. According to the oddball paradigm, non-target LEDs were alternated with the target in a pseudo-random sequence. The rows and the columns of the LEDs matrix were significantly intensified for 125 ms with 125 ms (power off) between intensifications (Figure 5). A stimulation sequence requires that all rows and all columns of the stimulation matrix illuminate at least once. Therefore, the acquisition of EEG data relative to two or more stimulation sequences is needed to correct the P300 component. The set of stimulations sequences delivered to the user represents a trial and allows selecting a target. A symbolic communicator (a sheet with 36 alphanumeric characters or icons, see Figure 6) can be overlaid to the stimulation panel based on the specific application in which the system is used.

### 2.2. Acquisition Module

The acquisition module represents the interface between the EEG acquisition device and the embedded hardware platform. This block, implemented on the sbRIO platform, provides communication with the EEG amplifier, relying on a protocol defined by the acquisition hardware. During the initialization phase, the acquisition block configures the external hardware; in particular, it selects the channel for the acquisition and decides whether to use the two digital lines as an external trigger. While operating, this block receives data from the EEG amplifier. It then retransmits them with appropriate markers/labels (i.e., Phase in Sequence, Stimulus Code, Stimulus Type) following the processing module. These labels will allow for a correct signal segmentation to extract control parameters and, generally, P300 detection. In addition, they provide information about the BCI session’s current state, allowing for the correct timing of the stimulation and information about the present stimulus intensified. In addition, the acquisition module is concerned with saving the data and its labels for further offline analysis. These data are stored in the memory available onboard the embedded system and are accessible through the FTP protocol.

### 2.3. P300 Signal Processing

**Figure 7:**
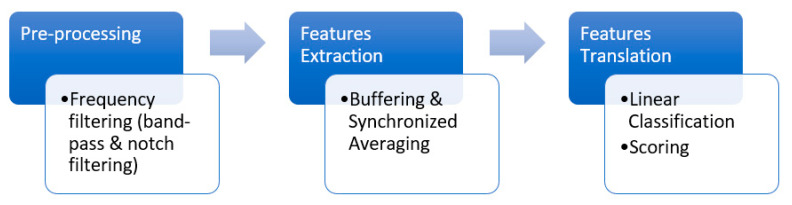
P300 signal-processing steps implemented on the FPGA and the real-time microprocessor.

All signal-processing operations needed for the extraction and classification of the P300 component were implemented on the embedded platform, exploiting its dyadic architecture based on FPGA and a real-time microprocessor. The main steps are illustrated in Figure 7.

The conditioning block is the first processing step, as reported in the previous BCI scheme. Next, the data coming from the acquisition module undergo frequency filtering operations to improve the signal-to-noise ratio. Frequency filtering includes a band-pass filter (0.2–80 Hz, Butterworth topology, with 40 dB/dec roll-off) that removes the continuous and high frequencies and a notch filter (with a 50 Hz cut-off frequency), which eliminates interferences from the power supply.

The feature extraction block extracts the evoked potentials from the continuous EEG signal. The P300 detection method followed the procedure developed by Farwell and Donchin. The EEG filtered signals coming from the eight channels are segmented in epochs beginning with the intensification and lasting for 800 ms: for each channel, an epoch was derived in association with each stimulus class, thus for each matrix row and column intensification. The method assumes that the epochs associated with the target stimulus will present a detectable P300, while the other epochs will not. Then, the epochs related to the target and non-target stimuli are averaged for each stimulus class over all stimulation sequences of a single trial for each electrode site. Equations (1) and (2) show the averaging calculations for each stimulus class (rows and columns):

R1mean = mean(R1(seq1), R1(seq2),.....R1(seqk)),
...... Rnmean = mean(Rn(seq1), Rn(seq2),.....Rn(seqk))(1)

C1mean = mean(C1(seq1), C1(seq2),.....C1(seqk)), ...... Cmmean = mean(Cm(seq1), Cm(seq2),.....Cm(seqk)),(2)

where R1(seq1) is, for example, the epoch relative to stimulus class 1 acquired during the first stimulation sequence. Therefore, R1mean, …, Rnmean are the averaged epochs relative to nth row stimuli and C1mean, …, Cmmean to the mth column stimuli. Particularly, R*_imean_* ∈ ℝ *^ns^^∗^^nch^* for i = 1, …, n and C*_imean_* ∈ ℝ *^ns^^∗^^nch^* for i = 1, …, m. ns is the total number of samples in a single epoch and nch the number of acquired channels, respectively 204 and 8.

The output of the features extraction block will be averaged features vectors (AveragedEpochs) related to each stimulus class and electrode site (Equation (3)).

AveragedEpochs = [R1mean,...Rnmean, C1mean,...Cmmean].(3)

The feature translator block translates the features vectors in a logic control signal independent of the specific application. It generally consists of linear classification. In particular, it receives as input the epochs averaged for each stimulus and the weights matrix, obtained through the offline analysis of previously acquired EEG data. Weights coefficients are extracted through an offline procedure based on the step wise linear discriminant analysis (SWLDA) [31]. Then, the feature translator block performs a linear combination of the features arrays (AveragedEpochs) with the weights (Weights), obtaining the scores vector (Y) for each stimulus class (Equations (4) and (5)).

Yrow1 = ΣAverageEpochs1(i, j) ∗ Weights(i, j), .... Yrown = ΣAverageEpochsn(i, j) ∗ Weights(i, j)(4)

Ycolumn1 = ΣAverageEpochsn + 1(i, j) ∗ Weights(i, j), .... Ycolumnm = ΣAverageEpochsn + m(i, j) ∗ Weights(i, j).(5)

Finally, the algorithm searches for the maximum scores (predictedrow and predictedcolumn) in the vector for the stimulus class relative to the rows (Yrow) and columns (Ycolumns) (Equation (6a–d)).

Yrow = (Yrow1, …, Yrown)(6a)

Ycolumn = (Ycolumn1, …, Ycolumnm)(6b)

predictedrow = argmax(Yrow)(6c)

predictedcolumn = argmax(Ycolumn).(6d)

The intersection of these values provides the winning item (selecteditem) (Equation (7)).

selecteditem = predictedrow ∩ predictedcolumn.(7)

### 2.4. BCI Operating Modes and Operator Interface

The BCI system implements two different operating modes: calibration and run modes. Calibration mode is needed to extract from acquired EEG data information for the classification in the online mode (run mode).

During the calibration mode, the system prompts the user with the character that he/she is expected to focus on (target); the user was asked to count the target stimuli during the visual stimulation. The BCI system acquires and saves EEG data and labels.

In the run mode, the user can choose the desired icon/letter on his/her paper communicator. Then, the embedded BCI starts the visual stimulation, and EEG data are transferred from the amplifier, saved, and processed. At the end of each trial, the classifier recognizes the winner stimulus and the BCI device communicates it to the user.

**Figure 8:**
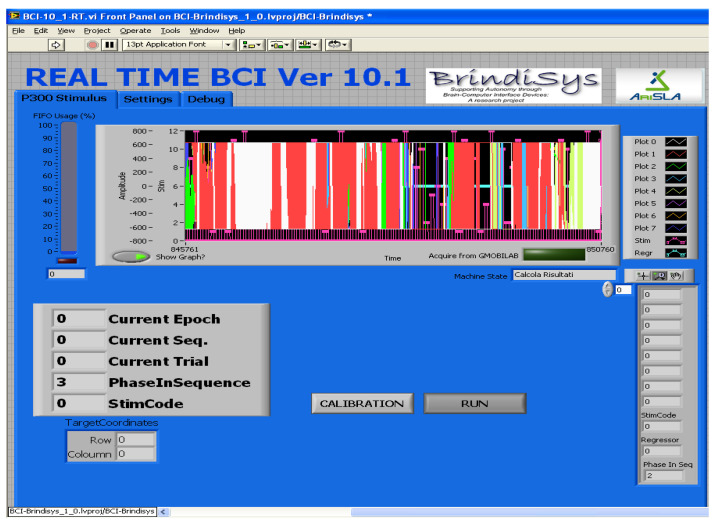
FPGA-embedded BCI interface: the operator can assess the correct functioning during the run phase, or he/she can manage the calibration phase.

**Figure 9:**
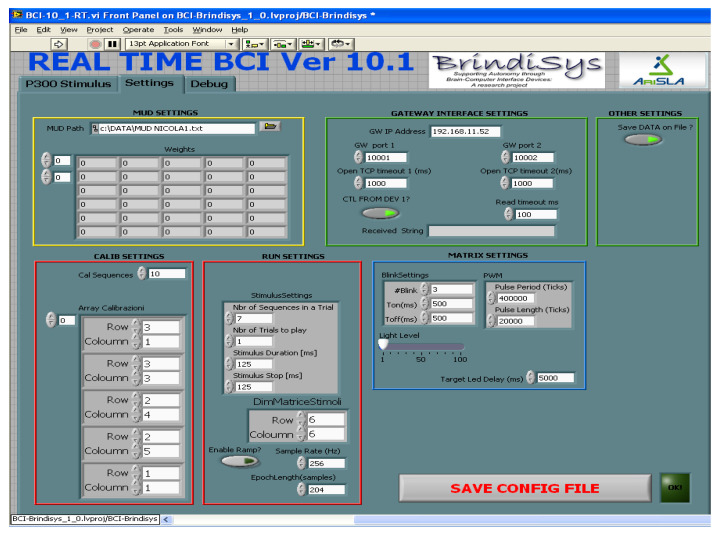
Operator interface of FPGA-embedded BCI that allows the configuration of the system.

BCI device is provided with a helpful operator interface to configure the BCI device and assess the correct functioning during the experimentation stage (Figure 8). In addition, the operator interface allows the configuration of some parameters relative to acquisition, processing, and stimulation (see Figure 9).

## 3. Results

**Figure 10:**
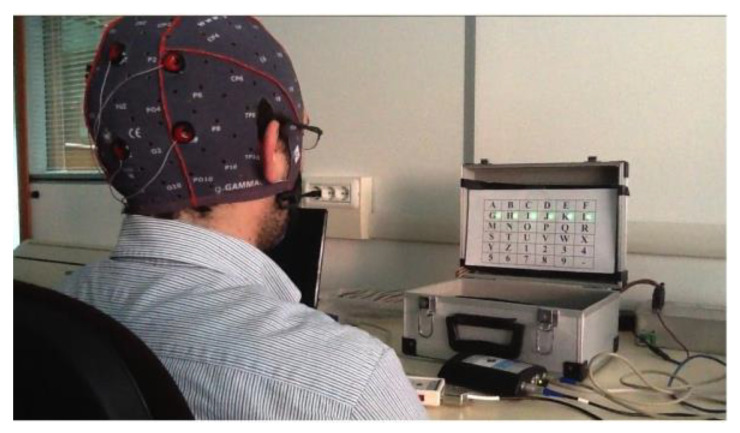
Experimental setup.

The system was initially tested on a healthy subject to verify the correct operation and evaluate its performance. The alphanumeric communicator has been overlaid on the stimulation panel to allow the user to perform a communication task. The subject was seated facing the stimulation panel (see Figure 10).

At first, the user was involved in the calibration phase: it delivered 10 stimulation sequences for 7 trials. Next, the system acquired and saved EEG data and labels for training the classifier in offline mode correctly.

After classifier training, the user could freely decide what icons the system had to recognize. The number of stimulation sequences in the run mode was lowered to seven. The user performed a relatively long session with 35 consecutive trials.

**Figure 11:**
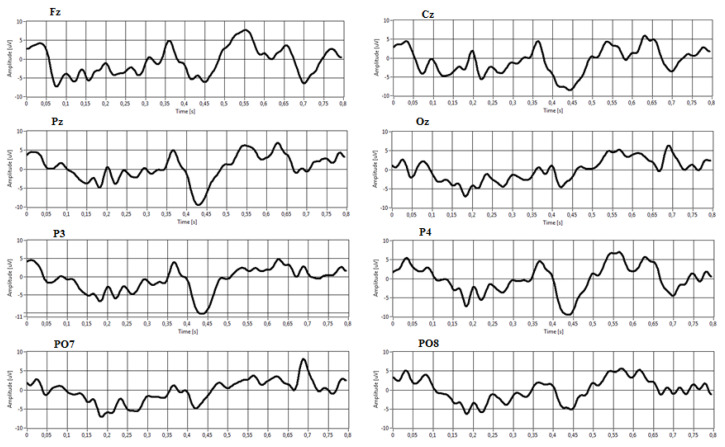
Examples of averaged target epochs.

**Figure 12:**
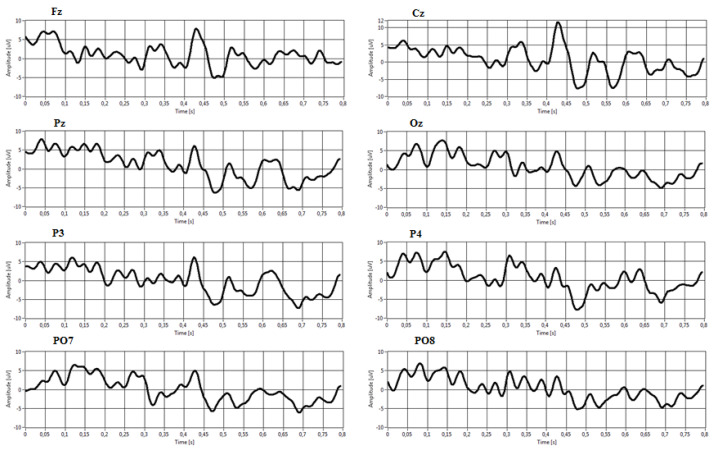
Examples of averaged non-target epochs.

Figure 11 and Figure 12 show an example of averaged target and non-target epochs extracted from the P300 Signal Processor relative to a target and non-target stimulus class during a trial. It is possible to note in Figure 8 the presence of a P300 event-related potential in the time interval around 350–400 ms from stimulus onset. This activation component becomes more negative in the electrodes Cz, Pz, P3, and P4.

To assess BCI performance, we evaluated accuracy and selection rate. In particular, Equation (8) defines accuracy parameter in percentage value:

Accuracy% = Hits/(Hits + Miss) ∗ 100,(8)

where Hits are the correct recognized targets and Miss is the wrong recognized target.

The system correctly recognized 21 targets, while the total targets were 35. Therefore it was obtained an accuracy of 60%. However, this result is encouraging as it has been obtained from a user entirely naive for the BCI protocol based on P300. Considering the 14 error cases, 11 refer to situations in which the system can identify at least the row or column relating to the target icon, 3 to situations in which the system fails. Naturally, the latter errors are due to the user’s distraction or fatigue.

**Figure 13:**
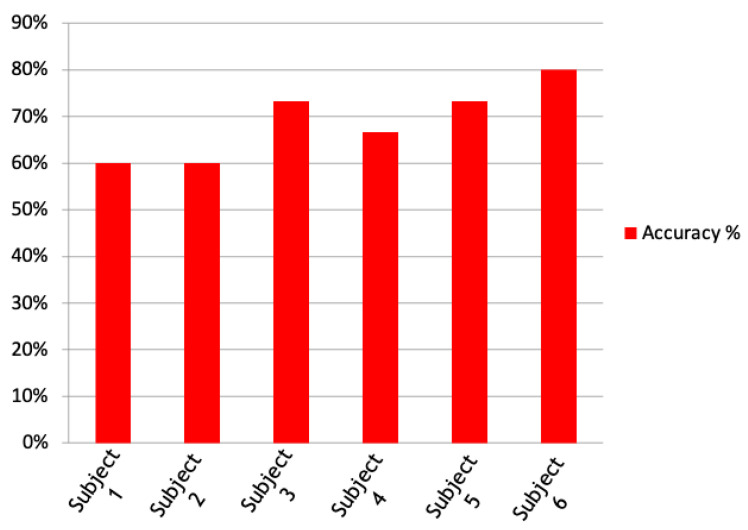
Accuracy results obtained from 6 different subjects involved in experimental sessions of 15 trials.

Then, the system was tested on six subjects that performed six sessions, each composed of 15 trials, with the alphanumeric communicator overlaid on the stimulation panel. The results in terms of accuracy are represented in Figure 13. The average accuracy is nearly 70%.

**Figure 14:**
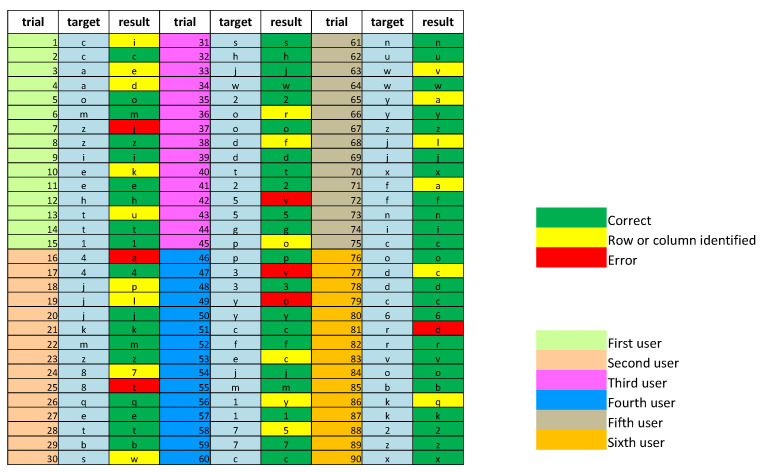
Detailed results obtained from 6 different subjects involved in experimental sessions of 15 trials.

Figure 14 gives details of the tests on the six subjects. It is worthing to point out that our system was tested on completely “illiteracy” users, and we reported results relative to their first sessions to demonstrate our system’s robustness. The columns labeled “Trial” show the identification codes of each trial: trials from 1 to 15 refer to the first subject; from 16 to 30 to the second subject; from 31 to 45 to the third subject; from 46 to 60 to the fourth subject; from 61 to 75 to the fifth subject; from 76 to 90 to the sixth subject. The “Target” columns indicate which icon the user was asked to fix. Finally, the “Results” columns show the system results.

The correct results are green; the wrong results are yellow and red. Specifically, considering the 28 cases of error, 22 instances refer to the situation in which the system can identify the row or column relating to the target icon (results in yellow), 6 in situations in which the system completely errs (results in red).

Equation (9) defines the selection rate, considering a minute as time unit:

SelectionRate = 60/SelectionTime.(9)

SelectionTime has been evaluated by using Equation (10):

SelectionTime = ST + PT + RT,(10)

where ST is the stimulation time, PT is the processing time (equal to 4 s), and RT is the results display time (5 s).

Stimulation time has been evaluated in the following way (Equation (11)):

ST = (SD + ISI) ∗ N_StimulusClasses ∗ N_StimulationSequences,(11)

where SD is the stimulus duration (125 ms), ISI is the inter-stimulus interval (125 ms), NStimulusClasses is the total number of stimulus classes, and NStimulationSequences is the total number of stimulus sequences (namely 12 and 7, respectively).

According to these data, the selection time is 30 s and, therefore, the selection rate of our BCI system is two letters per minute. It could be improved by reducing the number of stimulation sequences: indeed reducing the number of stimulation sequences from 7 to 5 and the results display time of some seconds, the selection rate changes from two letters per minute to three letters per minute.

## 4. Conclusions

A novel FPGA-embedded BCI system has been designed and implemented. In particular, a hardware FPGA-based system has been realized to achieve precise timing stimulation and efficient data processing. The system’s design is based on FPGA technology. The use of an FPGA-based device makes the system flexible because the functionality can be changed (so we have reconfigurable embedded hardware). Moreover, it is possible to implement parallel tasks that will be executed simultaneously and independently from each other, thus guaranteeing high performance (the hardware parallelism of the FPGA is exploited, which exceeds the computing power of the DSP). Since applications are implemented in hardware without an operating system, FPGA will run them reliably (so there will be no software-related time latencies). Moreover, the embedded portable platform avoids using bulky personal computers accompanied by commercial signal-processing software. The first P300 FPGA BCI system was illustrated in the work of [49], but only a simple filter was realized in the reconfigurable logic, while most of the processing was performed in softcore processors. Moreover, the system did not interface with the EEG amplifier. Instead, EEG data were sent from the PC using the TCP/IP protocol. Our system is a complete BCI system where EEG data are acquired using an EEG amplifier that interfaces with an FPGA-based system for pre-processing and P300 recognition.

This study’s significant shortcomings rely on the fact that the number of subjects trained and tested on this BCI system is limited. However, the primary objective of validating the functionality of the BCI is achieved. Another shortcoming of the present study is the offline calibration. To improve the usability of the system for end-users, an automatic online calibration procedure will be implemented. Moreover, the BCI performance (particularly, selection rate) could be improved by reducing the number of stimulation sequences or defining single-trial algorithms for P300 detection. Although the system is slow compared to conventional media, we must consider that the communication speed is less important than accuracy and reliability for users with disabilities. Furthermore, the problem of increasing the rate selection becomes less critical when the system is used in the domotic context since the user must select the icons less frequently.
